# Commentary on: Intralesional Tetracycline Injection, Pinch Technique, and Canthopexy for the Treatment of Severe Festoons: Preliminary Results

**DOI:** 10.1093/asjof/ojab058

**Published:** 2022-02-09

**Authors:** Salvatore J Pacella

**Affiliations:** Dr Pacella is the division head, Plastic and Reconstructive Surgery, Scripps Clinic Medical Group, Scripps M.D. Anderson Cancer Center, San Diego, CA, USA

The management of malar festoons represents one of the most challenging aspects of the lower eyelid and midface rejuvenation. In this article,^1^ the authors utilize a novel technique of serial tetracycline injection followed by pinch blepharoplasty and canthopexy. It is interesting to note that of the 15 patients in the study, only 12 went on to surgical intervention.

The successful management of malar deformities, particularly when coupled with the lower eyelid rejuvenation, can only be mastered by a comprehensive understanding of both the static and dynamic anatomy of this region (Video). The 3-dimensional (3D) ligamentous anatomy of the lower eyelid and midface is paramount to creating and implementing a successful strategy for patient treatment.^2^ It would benefit the text to include a description of the anatomy. The orbitomalar ligament (a.k.a. the orbicularis retaining ligament), which was originally described by Kikkawa et al in 1996,^3^ and later redescribed by Mendelson et al in 2002,^4^ is the critical ligamentous structure to identify during advanced open techniques. The orbitomalar ligament creates the floor of the lid-cheek junction, which creates the prominence of bulging orbital fat (ie, palpebral bags) along the arcus marginalis. The orbitomalar ligament also forms the roof of the prezygomatic space. The prezygomatic space is the malar compartment that contains retained fluid, fat, and excess skin, which contributes to the development of malar edema, malar bags, and festoons, respectively. The floor of the prezygomatic space is the zygomaticocutaneous ligament. These ligamentous attachments originate on the midface skeleton and insert ventrally in the subcutaneous plane.^5^ It is important to note that this space extends quite more laterally than most surgeons anticipate, which can often require surgical dissection beyond the confines of the orbit and midface to successfully correct the deformity.

Although the photographs do demonstrate minimal to modest improvement, the small series of patients is insufficient to make any profound conclusions about the utility of this technique for the treatment of severe festoons. In addition, the authors provide no validated instrument or questionnaire for patient satisfaction. They simply state that the patients had improved results. This, in my opinion, is an insufficient conclusion.

The 3D potential space in the malar region is the foundation of understanding comprehensive rejuvenation of the malar region. It is also implicated as the methodology for the failure of most minimally invasive treatments. The technique described does not reconfigure the anatomic constraints that create festoons. In my opinion, the technique is insufficient for aesthetic contouring.
